# Spontaneous Coronary Artery Dissection in Acute Coronary Syndrome: Report of a Series of Cases with 17 Patients

**DOI:** 10.5935/abc.20160170

**Published:** 2016-11

**Authors:** Ana Rita Godinho, Mariana Vasconcelos, Vitor Araújo, Maria Júlia Maciel

**Affiliations:** Hospital de São João, Porto - Portugal

**Keywords:** Acute Coronary Syndrome, Dissection, Coronary Aneurysm, Coronary Angiography

## Introduction

Spontaneous Coronary Artery Dissection (SCAD) has prevalence of 0.2 to 1.1% of the total angiograms performed for Acute Coronary Syndrome (ACS)^1,2^ and mostly affects young women.^3^ The etiology is not fully known,^2,3^ but the term "spontaneous" excludes all dissections associated with interventions or trauma.^1^ Treatment varies from the conservative approach to revascularization,^4^ with a favorable long-term prognosis.^5^

This study aimed to assess the characteristics, the clinical presentation, therapeutic approach and follow-up of cases of SCAD with ACS presentation during a period of 7 years.

## Methods

This is a descriptive and retrospective analysis of patients admitted to a Cardiology Service for 7 consecutive years, between 2008 and 2014, with a diagnosis of ACS due to SCAD. The diagnosis of SCAD was attained in the presence of angiographic characteristics obtained through coronary angiography.

Data were obtained from medical records of hospitalization and subsequent consultations.

## Results

Of the 4,600 patients admitted with ACS at the Cardiology Service, 17 patients (0.4%) had SCAD as the cause. The mean age was 51 ± 9 years; ten patients were women, of which 5 were in the postmenopausal period.

SCAD may have been related to oral contraceptive use in three cases, intense exercises in one case, and smoking in eight cases.

All patients had a diagnosis of acute myocardial infarction (AMI), 59% with ST-Segment Elevation (STEMI) and 41% with non-ST-segment elevation (NSTEMI).

Most progressed with Killip class I (94%), with a median troponin I value of 10 [P25 3] ng / dL. Left ventricular systolic function was preserved in 82% and only one patient developed severe systolic dysfunction.

**Figure 1:**
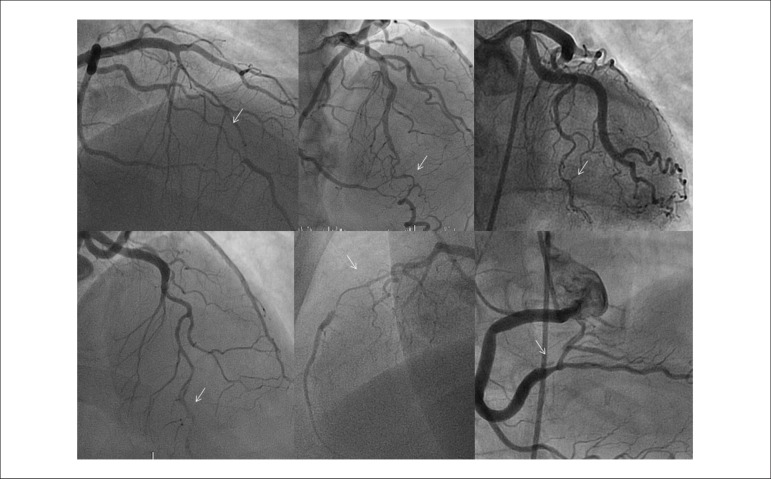
Coronary angiography images showing spontaneous coronary dissections diagnosed in the context of acute coronary syndrome.

During hospitalization, which lasted 10 ± 5 days, all patients underwent coronary angiography (Figure 1), with only one artery with SCAD being identified in each patient. All SCDs were classified as type 2 angiographic variant. The Anterior Descending artery (ADA) was the most frequently affected (seven patients), followed by the the right coronary artery (RCA) (five patients), while four patients had dissection of the circumflex artery (Cx) and one patient had dissection of the left main coronary artery (LMCA).

Four patients had complications during the hospital stay: one patient with an episode of atrial fibrillation, three with reinfarction and one with associated pericarditis. All reinfarction cases were submitted to coronary angiography, which showed progression of the SCAD with occlusion of the distal vessel; a conservative approach was instituted. 

All hospitalized patients that had, at the catheterization, Thrombolysis In Myocardial Infarction (TIMI) flow 3 in the affected artery were treated with medical therapy: dual antiplatelet therapy, heparin and statins. One patient underwent angioplasty with bare-metal stent implant for occlusion of the artery showing dissection (CD). In three other cases, concomitant atherosclerosis was identified; two patients underwent angioplasty with covered stent implantation and one underwent surgical revascularization. 

At the median follow-up of 52 [P25 30] months, only one patient had a new atherosclerotic AMI documented by coronary angiography.

Of the patients who underwent control coronary angiography (47%), all showed dissection resolution; 76% of the patients, asymptomatic, were submitted to non-invasive ischemia testing (nine performed stress test; four, perfusion scintigraphy - three of which also performed stress test and one patient underwent cardiac MRI perfusion), which was negative.

During the follow-up no deaths were recorded and no patient developed heart failure (Table 1).

**Table 1:** General characteristics of clinical presentation and follow-up

Patient	Gender	Age	AMI	Triggering factors	Artery	Complications in the acute phase	Treatment	LVF	Follow-up (52 [P25 30] months)
1	M	64	STEMI	Tobacco	RCA	No events	PCI	Preserved	No events
2	M	41	NSTEMI	Absent	RCA	No events	Conservative	Preserved	No events
3	F	55	NSTEMI	Absent	RCA	No events	Conservative	Slight	AMI
4	F	38	STEMI	OCs	CX	No events	Conservative	Preserved	No events
5	M	59	STEMI	Tobacco	CX	AF	PCI	Preserved	No events
6	F	60	NSTEMI	Absent	RCA	No events	Conservative	Preserved	No events
7	F	47	NSTEMI	Absent	ADA	No events	Conservative	Preserved	No events
8	F	60	NSTEMI	Absent	CX	Reinfarction	Conservative	Preserved	No events
9	F	37	NSTEMI	OCs	RCA	No events	Conservative	Preserved	No events
10	F	49	NSTEMI	Absent	CX	Reinfarction	Conservative	Preserved	No events
11	M	54	STEMI	Absent	ADA	Pericarditis + reinfarction	Conservative	Moderate	No events
12	F	50	STEMI	Exercise + tobacco	ADA	No events	Conservative	Slight	No events
13	M	59	STEMI	Tobacco	ADA	No events	CABG	Preserved	No events
14	M	48	STEMI	Tobacco	LMCA	No events	Conservative	Preserved	No events
15	F	63	STEMI	Tobacco	ADA	No events	Conservative	Preserved	No events
16	M	39	STEMI	Tobacco	ADA	No events	PCI	Preserved	No events
17	F	50	STEMI	OCs + tobacco	ADA	No events	Conservative	Preserved	No events

AMI: acute myocardial infarction; LVF: left ventricular function; M: male; F: female; STEMI: acute myocardial infarction with ST-segment elevation; RCA: right coronary artery; PCI: percutaneous coronary intervention; NSTEMI: acute myocardial infarction without ST segment elevation; F: female; OCs: oral contraceptives; CX: circumflex artery; AF: atrial fibrillation; ADA: anterior descending artery; CABG: coronary artery bypass grafting; LMCA: left main coronary artery.

## Discussion

The SCAD often affects young individuals between 35 and 40 years of age.^5^ Approximately 70% are women and in 30% of cases, the SCAD is related to pregnancy.^3^ It has a broad clinical presentation spectrum.^5^

Its etiology/pathophysiology is not completely understood, but it is related to atherosclerotic disease; the peripartum period; connective tissue disease; vasculitis; smoking; oral contraceptives; SAH; cocaine use; coronary vasospasm; cyclosporine and intense exercise. Recently, a close association has been identified between SCAD and the presence of fibromuscular dysplasia, and, therefore, its presence should be ruled out.^2,3,6-8^

It can affect one or more coronary arteries, being more frequent in the ADA.^3^ RCA dissections are more common in men, while left main coronary artery dissections are more common in women.^9^

Its identification is often difficult,^10^ and a high degree of clinical suspicion is essential. The use of complementary techniques such as the intravascular ultrasound (IVUS) and coronary computed tomography (CT) contributes to a better identification and classification of SCAD. Coronary CT is the most sensitive technique for the diagnosis of SCAD due to a higher resolution, but lower penetration than the IVUS. Although the coronary angiotomography allows the assessment of atherosclerotic lesions, it has limited usefulness in SCAD due to its low spatial resolution.^3,10,11^

Treatment varies according to the site of dissection, the number of involved vessels, distal flow, the hemodynamic status of the patient and the possibility of intervention.^4^

In stable patients with normal coronary flow, the treatment is preferably the conservative approach.^4,5^ Angioplasty is indicated only in cases of ischemia and one-vessel disease, due to the high risk of propagation of the dissection related to the procedure;^4,12^ there is still no ideal stent for the treatment of such lesions.^4^ Surgical revascularization is appropriate in multivessel disease or when the LMCA is affected.^4^ The risk of the procedure is related to the non-identification of the true lumen when performing CABG.^9^ Fibrinolysis is not recommended due to the risk of SCAD propagation.

After the acute phase, the estimated survival is 70 to 90%.^5^ There is risk of recurrence in 50% of the patients, which leads us to consider the presence of a systemic susceptibility to dissections, evidenced by the initial event.^6,7,13^

In short, we emphasize the need to consider the possibility of SCAD in middle-aged women, presenting with AMI, often without identifying the triggering factor. It is believed that the conservative approach is the most appropriate and, therefore, it was the one most widely used. As for the follow-up of these patients, although usually a long-term one, it is poorly defined. In this registry, most complications were observed during hospitalization. The long-term behavior was relatively benign.

It is therefore essential to develop a homogenous approach for the follow-up of these patients. 

## Conclusion

Spontaneous coronary artery dissection is a rare differential diagnosis should be considered in the presence of acute coronary syndrome. This entity requires the ruling out of possible associated systemic pathologies and a targeted therapeutic approach, being correlated with a favorable long-term prognosis.
